# 623. Catastrophic Electrical Injuries Tied to Copper Theft in the Homeless: Clinical and Policy Considerations

**DOI:** 10.1093/jbcr/irag033.449

**Published:** 2026-04-06

**Authors:** Albert A Ávila Álvarez, Jenny Carolina Paez Figueroa, Martina Llinás Tono, Diego Hernando Romero Pinzon, Luz Adriana Caro Santos, Norberto Navarrete Aldana, Gabriela Castro Gómez, Ludwin Ferney Rojas Espinosa

**Affiliations:** Subred Integrada de servicios de Salud Norte ESE-Hospital Simón Bolívar, Bogotá, Distrito Capital de Bogota; Subred Integrada de servicios de Salud Norte ESE-Hospital Simón Bolívar, Bogotá, Distrito Capital de Bogota; Subred Integrada de servicios de Salud Norte ESE-Hospital Simón Bolívar, Bogotá, Distrito Capital de Bogota; Subred Integrada de servicios de Salud Norte ESE-Hospital Simón Bolívar, Bogotá, Distrito Capital de Bogota; Hospital Simón Bolívar, Bogotá, Distrito Capital de Bogota; Subred Integrada de servicios de Salud Norte ESE-Hospital Simón Bolívar, Bogotá, Distrito Capital de Bogota; Subred Integrada de servicios de Salud Norte ESE-Hospital Simón Bolívar, Bogotá, Distrito Capital de Bogota; Universidad del Rosario-Fundación Santa fe de bogotá, Bogotá, Distrito Capital de Bogota

## Abstract

**Introduction:**

High-voltage electrical injuries (≥1000 V) cause devastating outcomes, including rhabdomyolysis, amputations, and death. In Colombia, copper’s high market value (~$278 M exports in 2023; $7.50–$8.00/kg locally) has fueled systematic cable theft. This not only disrupts essential services and creates multimillion-dollar losses but also inflicts catastrophic trauma—most often on homeless individuals with substance use disorders. Similar patterns are seen globally in impoverished settings. We propose a syndemic framework, where poverty, homelessness, SUD, and electrical trauma converge to amplify harm. Addressing this crisis requires integrated action across health, social policy, infrastructure, and legal regulation.

**Methods:**

Retrospective series (January 2024–June 2025) of 15 homeless individuals with electrical burns treated at a referral burn center in Bogotá. Demographic, clinical, and outcome variables were collected and analyzed using descriptive statistics with Wilson 95% confidence intervals.

**Results:**

Males accounted for 93.3% (14/15; 95%CI 70.2–98.8), with a mean age of 33.1 ± 8.4 years. High-voltage injuries represented 86.7% (13/15; 62.1–96.3). The median %TBSA was 11% (IQR 4.5–16). Rhabdomyolysis occurred in 40.0% (6/15; 19.8–64.3), with peak CPK values reaching 50 330 U/L. Acute kidney injury (AKI) was observed in 6.7% (1/15; 1.2–28.3). Amputation was required in 13.3% (2/15; 3.7–37.9). In-hospital mortality was 20.0% (3/15; 7.0–45.2). Notably, 100% of the patients had a documented substance use disorder (SUD).

**Conclusions:**

We identified a vulnerable cohort of homeless men with substance use disorders (SUD) who sustained predominantly high-voltage (HV) electrical burns. The convergence of HV trauma, social vulnerability, and SUD explains the severe outcomes observed. Mortality reached 20%—well above reported ranges of 2–12%—while amputations (13.3%) reflected extensive HV tissue and vascular damage. Rhabdomyolysis occurred in 40% of cases, and all patients had SUD. Copper theft has been consistently linked to fatal electrocutions in Colombia and other low- and middle-income countries, paralleling South Africa’s Izinyoka phenomenon. Framing this as a syndemic highlights the need for integrated responses across public health and legal systems.

**Applicability of Research to Practice:**

This study identifies a syndemic of homelessness, substance use disorder (SUD), and economic precarity fueling catastrophic electrical injuries linked to copper theft. Outcomes are dire—rhabdomyolysis 40%, amputations 13.3%, mortality 20%—with immense social costs.

Action Priorities.

Public Health: Outreach programs offering SUD treatment, mental health care, and standardized protocols for high-voltage injuries.

Scrap Market Control: Enforce seller registration, traceability, and cash restrictions.

Infrastructure: Replace copper wiring with alternatives like fiber optic, proven effective in Bogotá.

**Funding for the study:**

N/A.

